# VIRMISCO – The Virtual Microscope Slide Collection

**DOI:** 10.3897/zookeys.741.22284

**Published:** 2018-03-07

**Authors:** Peter Decker, Axel Christian, Willi E.R. Xylander

**Affiliations:** 1 Senckenberg Museum of Natural History Görlitz, Görlitz, Germany

**Keywords:** Database, digitisation, morphology, museum collection, soil invertebrates, type material, visualisation

## Abstract

Digitisation allows scientists rapid access to research objects. For transparent to semi-transparent three-dimensional microscopic objects, such as microinvertebrates or small body parts of organisms, available databases are scarce. Most mounting media used for permanent microscope slides deteriorate after some years or decades, eventually leading to total damage and loss of the object. However, restoration is labour-intensive, and often the composition of the mounting media is not known. A digital preservation of important material, especially types, is important and an urgent need. The Virtual Microscope Slide Collection – VIRMISCO project has developed recommendations for taking microscopic image stacks of three-dimensional objects, depositing and presenting such series of digital image files or z-stacks as an online platform. The core of VIRMISCO is an online viewer, which enables the user to virtually focus through an object online as if using a real microscope. Additionally, VIRMISCO offers features such as search, rotating, zooming, measuring, changing brightness or contrast, taking snapshots, leaving feedback as well as downloading complete z-stacks as jpeg files or video file. The open source system can be installed by any institution and can be linked to common database or images can be sent to the Senckenberg Museum of Natural History Görlitz. The benefits of VIRMISCO are the preservation of important or fragile material, to avoid loan, to act as a digital archive for image files and to allow determination by experts from the distance, as well as providing reference libraries for taxonomic research or education and providing image series as online supplementary material for publications or digital vouchers of specimens of molecular investigations are relevant applications for VIRMISCO.

## Introduction

Recent advantages in digitisation facilitate use, processing, duplication, distribution, archiving, and playback on common media devices, and improved applications for inquiries and comparison. Furthermore, digital copies protect the originals or serve as documentation in case of loss or damage.There are many strategic initiatives to digitise collection material from natural history museums, such as ultra-high resolution images of e.g., insect boxes, labels or three-dimensional scans (e.g., skulls, taxidermy mounts) (Mantle et al. 2012, Blagoderov et al. 2012, Holovachov et al. 2014, Copes et al. 2016, Short et al. 2018). While projects on virtual microscopy in biology or micropalaeontology are rare, in medical applications virtual microscopy and virtual (histological) slide collections are well known and acknowledged as beneficial e.g., for documentation, teaching, diagnoses, and research (Kumar et al. 2004, Gu and Ogilvie 2005, Helin et al. 2005, Krippendorf and Lough 2005, Goldberg and Dintzis 2007, Mikula et al. 2007, Dee 2009, Weinstein et al. 2009). However, these are mostly restricted to two-dimensional histological slices or cell biology, are not open source, are not accessible publicly online, restricted to a specific manufacturer or do not comply with the needs of a soil zoological collection.

Permanent microslides in collections often lose their quality due to ageing and physico-chemical alterations of the mounting media. Whereas some mounting media like Canada balsam, Euparal and glycerol-paraffin sealed with Glyceel show proper quality even after 50 to more than 150 years, others darken or deteriorate by dehydration, contraction, oxidation, or crystallisation of the media (Brown 1997, Brown and De Boise 2006, Allington and Sherlock 2007, Neuhaus et al. 2017). Additionally, the former collection owners may have used several different mounting media or the composition was changed by the producers or manufacturers in the course of time. Thus, the mounting media are not known for all slides. Cleaning, re-mounting and restoration is very labour-intensive or even impossible for some mounting media. Furthermore, the object may be damaged during the re-mounting process (Upton 1993, Brown and De Boise 2006, Neuhaus et al. 2017). In practice, objects are already irretrievably damaged or partly destroyed when alterations are noticed by the curator. Several microscope slide collections have already been lost or will be lost within the next decades (Upton 1993, Jersabek et al. 2010, Lillo et al. 2010, Neuhaus et al. 2017).

Unfortunately, most institutions and collections do not have staff with experience on microscope slide restoration and no financial resources for this time-consuming task. Often the general storage and conservation conditions of the collection (e.g., temperature, humidity, light exposure, volatiles) require replacement or new investments (e.g., cabinets).

To rescue at least the relevant information of the valuable collection specimens an equivalent digital documentation, especially of type material, is crucial. However, digital images can never replace the original specimen and restoration of a microscope slide collection should be the first goal of a curator or institution.

In this publication we introduce the open source system “the Virtual Microscope Slide Collection – VIRMISCO” to present digital microscope images of different focal planes. General recommendations for digitisation of three-dimensional collection objects on permanent microscope slides of soil fauna and other small organisms are also provided.

**Figure 1.**
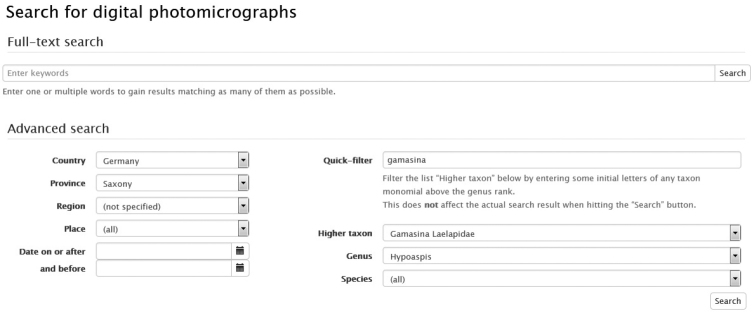
Screenshot of the VIRMISCO “Search” page.

## Objectives

The **Vir**tual **Mi**croscope **S**lide **Co**llection – VIRMISCO project at the Senckenberg Museum of Natural History Görlitz aims to develop recommendations for methods and procedures for digitisation of three-dimensional microscope objects and to make their information accessible online for free public use (open access database). Such an (almost) complete documentation with digital images of microscopic objects guarantees the permanent conservation at least of the relevant taxonomic information of most of the objects. Within VIRMISCO recommendations for light microscopic documentation of slide mounts were developed and a wide range of microscopic methods (e.g., phase contrast, differential interference contrast, confocal microscopy, digital microscopy) were tested in order to receive optimal results. Digital image files are made available by an online database via an open access internet platform. Metadata of specimens, collections, localities, sampling and the production of image sets are provided to optimise search and retrieval of the data in the internet.

Specimens, types or voucher for which molecular data are available, e.g., via Barcode of Life Data Systems (BOLD) or/and GenBank, documented with digital images may be deposited on the VIRMISCO platform. Such correlation of morphological and genetic information fulfils the demands of integrative taxonomy. Within the last three years, specimens or permanent microscope slides (including type material from more than 400 species) of various taxa of soil fauna (Acari, Collembola, Protura, Myriapoda, Plathelminthes, Tardigrada, Nematoda) from the collections of the Senckenberg Museum of Natural History Görlitz (SMNG) have been digitised (Table 1).

**Table 1.** Number of digitised specimens, taxa and types available online in VIRMISCO (1 February 2018).

	**Taxa**	**Specimens**	**Types**
**Gamasina**	87	215	198
**Uropodina**	34	116	116
**Oribatida**	5	15	1
**Collembola**	23	58	21
**Tardigrada**	2	2	0
**Protura**	3	4	0
**Diptera**	1	1	0
**Myriapoda**	21	100	18
**Nematoda**	1	2	0
**Plathelminthes**	5	11	0

The open source internet platform comprises a viewer and the digitised material may be employed for a wide range of applications: (1) Digital preservation of important or fragile material, especially of type material, to avoid loss or damage e.g., during loan; (2) archival of digital image files from collections or project data to verify taxonomic information; (3) determination from the digital images by experts; (4) creation of a reference library for taxonomic research or education; (5) provision of image series of type specimens (digitypes) or supplementary information for publication (e.g., series of specimens); (6) provision of image series of voucher specimens (or type material) for which molecular data is available.

The VIRMISCO system offers features such as interface to link it to other databases, search functions, rotating, zooming, measuring (two- or three-dimensional), changing brightness or contrast, taking snapshots, leaving feedback, downloading complete z-stacks as jpeg files or video file as well as a wide range of metadata fields on the collection object and the technical data (e.g., used camera, microscope, settings).

## Implementation

The open source system VIRMISCO can be set up individually by any institution on a server. It can be linked to or integrated in an already existing system of databases or data warehouses using the featured interfaces. Other modifications, adaptations or upgrading according to the individual needs are possible.

## Search engine

A search engine provides a full-text search. Advanced searches are also possible, e.g., hierarchical availability for locality (country, province, region, place), filtering of a taxon with hierarchical order (higher taxon, genus, species) and date or period of sampling.

**Figure 2.**
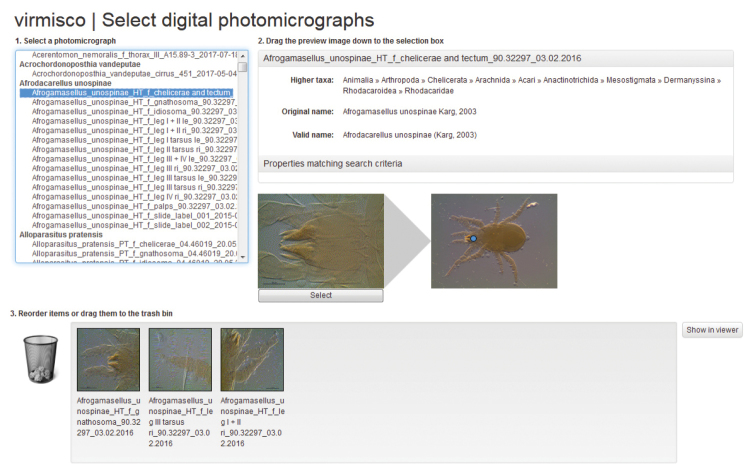
Screenshot of the VIRMISCO “Results” page.

Image stacks that meet the criteria specified by the query are grouped by objects (Fig. 2). General information about the selected object is displayed. An (animated) preview of the image stack video is visible and (if available) an overview image with a marking of the digitised part of the object the image stack refers to. The selected objects to be shown in the viewer can be sorted or removed.

## Viewer

**Figure 3.**
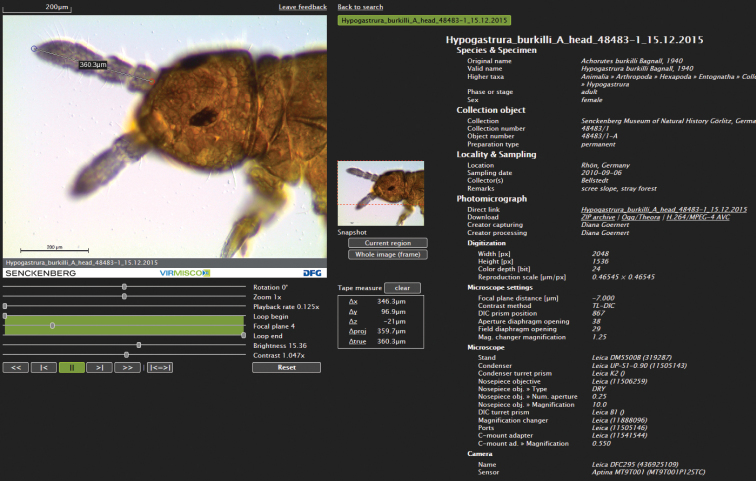
Screenshot of the VIRMISCO “Viewer”.

The core of the viewer (Fig. 3) is the image display, where an image or image series are presented (OGG/Theora file). The display area can be moved by dragging. On a thumbnail, the current display area of the image display is indicated in the total image with a snap frame.

**Functions**: Several functions and features are available to control and modify the image video file, e.g., rotation, zoom, playback rate, brightness and contrast (the latter two only when paused). The user can choose any focal plane or set the start and end of the playback loop. Common media control buttons are available, e.g., pause, play forward/backward, skip to start/end, and endless loop.

Body parts or regions, e.g., tarsus or chaeta, can be measured when the magnification scale of the image or series is available. By choosing any two points on one or two different focal planes, the distances between the different axes (vertical = X, horizontal = Y, height = Z) and/or the distance (∆true) between two points is indicated in the viewer. Snapshots of the currently displayed modified or whole image (the video frame) can be saved in a new frame.

**Download images/videos**: Image stacks as ZIP archive (JPEG) or in two video formats (OGG/Theora or H.264/MPEG-4 AVC).

**Metadata**: Information and metadata on the digitised object, image, and the equipment used is provided, e.g., taxon, collection, locality, sampling, microscope slide, settings, microscope, and camera used.

**Feedback**: Visitors can leave a message or feedback with reference to an actually viewed image stack using a contact form.

## Editor

Input of data and image stacks is done via an input form. Authorised users can import their biological and technical metadata offline and upload them later. This may be done manually or by using the import function.

Metadata fields: More than 20 information fields are currently available for each object (e.g., species, specimen, collection object, locality, sampling) and more than 80 values for each photograph (digitisation, microscope settings, microscope, camera, exposure settings, and histogram adjustments). According to the needs fields can be added or renamed.

**Import**: Technical metadata from a microscopes slide object are imported on a local server via a web-based form or by uploading the LAS files generated by Leica microscopic camera systems (image stack and technical metadata).

Alternatively cooperation with the Senckenberg Museum of Natural History Görlitz can be considered to upload image stacks with the associated metadata of specimens to the Görlitz VIRMISCO system. The provider of the image files and data may choose a Creative Common (CC) license that condition the terms of use.

**Conversion of images routine**: TIFF-files are used as master file in VIRMISCO. The TIFF-files are automatically converted to JPEG and subsequently converted to final derivatives by an implemented conversion routine: ZIP archive with JPEG files, OGG/Theora and H.264/MPEG-4 AVC. In the JPEG files a footer with relevant information (e.g., direct link, species, collection number, institution logo) on the object is included. The conversion routine is scheduled for one time per day (midnight). Thus, derivatives are available one day later.

## Inventory of digitised objects at SMNG

VIRMISCO currently (1 February 2018) provides more than 4,700 image stacks of about 180 taxa and more than 350 types, basically from collection material of the Senckenberg Museum of Natural History Görlitz (Tab. 1).

## Time required for digitisation

Once familiar with the microscoping technique and the VIRMISCO user interface it takes approximately one hour to take a 15 z-stack series of one specimen of Acari and import it into VIRMISCO. However, time depends on investigated taxa and used microscope systems. Storage space of all files amounts currently 1.1 TB, including original TIFFS, derivatives (videos), and total views.

## Recommendations for digitisation

The authors experiences in digitizing SMNG collection material, the comparison of different light microscopy types and feedback from colleagues of various institutions and different fields of biology or physics add up to general recommendations to be considered when digitizing or planning digitisation projects of permanent microscope slide mount collections of three-dimensional objects, especially soil organisms.

The photographic and microscopic equipment used depends on the fitting, needs and budget of the institution and the specific imaging demanded for certain taxa or characters. Generally, good results can be achieved with bright-field microscopy. Differential interference contrast microscopy (DIC) shows very good results regarding lateral contrast. To capture three-dimensionality of an object a series of images at different focal planes should be taken. If available a digital motor-focus or macro-rail should be used to define the focus distances between the single images. Auto-montage images or focus stacks bear the risk of incorrect software calculations sometimes producing erroneous final montage-images or artefacts when applied to transparent or semi-transparent objects or using DIC microscopy (Neuhaus et al. 2017).

Non-compressed image files, e.g., TIFF format, are recommended as a common master image file. For subsequent size comparison or measurements a scale bar with the used linear measure labelled should be embedded into each image. Metadata on the taxon, specimen, collection, inventory number, type status, sex, and other label information (locality, sampling) must be provided for each image series. Furthermore, information on the documented part (e.g., body region), if not using a total view only, and the view of the object (e.g., ventral, lateral) is indispensable. Technical information like the microscope, camera and microscope settings used are helpful for documentation and data re-usability. An overview image of the object and/or the complete microscope slide documents and correlates original label information and gives a quick impression of the condition of the object or microscope slide. The expertise of an experienced taxonomist for the group investigated is crucial to select the characters of taxonomic relevance for documentation for digitisation. A single total view is usually not useful to document and preserve the information of the taxonomic characters of an object or type specimen.

## Outlook

The Senckenberg Museum of Natural History will continue to digitise important material (especially type material of soil organisms) and import existing series or image files (e.g., whole slide photographs). Other institutions are invited for a wide range of cooperation, e.g., to modify or upgrade VIRMISCO or to present their images on the VIRMISCO system of the SMNG. Updated versions will be available online on GitHub.

## Project Information

Project title: Development of standards for the photographic documentation of permanent microscope slide mounts in precarious mounting media. The photographs are available on the internet platform “VIRMISCO – The Virtual Microscope Slide Collection”.

**Funding**: The project was funded by the DFG (XY 12/6-1) from May 2014 to December 2017.

**Personnel**: The project was conducted at the Senckenberg Museum of Natural History under the supervision of Willi Xylander and Axel Christian. Eberhard Wurst (2014–2016) and Peter Decker (2016–2017) were involved as project managers. Diana Goernert (2014–2017) and Kerstin Franke (2014-present) provided technical assistance.

**Design and Software**: SednaSoft A. Schaffhirt and A. Wünsche GbR, Biesnitzer Straße 8, 02826 Görlitz, Germany and Senckenberg Museum of Natural History Görlitz.

**Email address**: virmisco@senckenberg.de

**Suggested citation of VIRMISCO**: Christian, A., Decker, P., Wurst, E. and W.E.R. Xylander: VIRMISCO – The Virtual Microscope Slide Collection. www.virmisco.org.

**Microscope equipment used at SMNG**: Leica DM5500B DIC microscope and Leica M165C stereomicroscope, both with Leica DFC295 camera.

**Integration to other databases**: All available digitised objects in the GBIF database on soil zoology, “Edaphobase” (http://www.edaphobase.org, see Burkhardt et al. 2014) are linked to the respective images series in the SMNG VIRMISCO.

## Manual

A manual for the VIRMISCO Search, Results, and Viewer pages is available online http://cms.virmisco.org/index.php/manual.html

## Web location (URIs)

**Homepage**: http://www.virmisco.org

**Project description SMNG**: http://www.senckenberg.de/root/index.php?page_id=18729

**Project description DFG**: http://gepris.dfg.de/gepris/projekt/248331536?language=en

## Repository

**Repository Type**: GitHub.

**Browse URI**: https://github.com/virmisco/virmisco

Source code: CC0 1.0 Universal (CC0 1.0) Public Domain Dedication.

## Terminal equipment

**Display**: All control elements are accessible and all represented information is visible from 1,000 pixel width and 660 pixel height.

**Browser**: VIRMISCO can be used with almost every common computer browser, but had been optimised for Mozilla Firefox (v. 44/45.2), Microsoft Internet Explorer (v. 8), and Microsoft Edge (v. 38).

**System Requirements**: Fast internet connection and a mass storage are required.

**Programming languages**: JavaScript, HTML, PHP, shell script, SQL.

**Utility software**: Apache HTTP Server, Redis, MariaDB.

**External frameworks**: Behat (v. 2.5.5), Behat MinkExtension (v. 1.3.3), Behat MinkGoutteDriver (v. 1.1.0), Fabpot Goutte (v. 1.*), PHP Markdown (v. 1.6.0), Predis (v. 1.0.1). Composer file for automatically installing frameworks available in the GitHub repository https://github.com/virmisco/virmisco.

## Application programming interface

The data collected in the database are accessible as XML documents at any time. For this purpose, an HTTP-based data provider is used as the OAI-PMH, which uses METS as a container format. DarwinCore (including expedient extensions) is also to be used as a metadata format.
